# Analytics, Properties and Applications of Biologically Active Stilbene Derivatives

**DOI:** 10.3390/molecules28114482

**Published:** 2023-06-01

**Authors:** Mariusz Kluska, Joanna Jabłońska, Wiesław Prukała

**Affiliations:** 1 Faculty of Sciences, Siedlce University of Natural Sciences and Humanities, 3 Maja 54, 08-110 Siedlce, Poland; joanna.jablonska@uph.edu.pl; 2 Faculty of Chemistry, Adam Mickiewicz University, Uniwersytetu Poznańskiego 8, 61-614 Poznań, Poland; wprukala@amu.edu.pl

**Keywords:** analytics, biological activity, stilbene derivatives, preservatives, stability

## Abstract

Stilbene and its derivatives belong to the group of biologically active compounds. Some derivatives occur naturally in various plant species, while others are obtained by synthesis. Resveratrol is one of the best-known stilbene derivatives. Many stilbene derivatives exhibit antimicrobial, antifungal or anticancer properties. A thorough understanding of the properties of this group of biologically active compounds, and the development of their analytics from various matrices, will allow for a wider range of applications. This information is particularly important in the era of increasing incidence of various diseases hitherto unknown, including COVID-19, which is still present in our population. The purpose of this study was to summarize information on the qualitative and quantitative analysis of stilbene derivatives, their biological activity, potential applications as preservatives, antiseptics and disinfectants, and stability analysis in various matrices. Optimal conditions for the analysis of the stilbene derivatives in question were developed using the isotachophoresis technique.

## 1. Introduction

The structure of the stilbene molecule was first described in 1829 by A. Laurent. Since then, many different stilbene derivatives have been synthesized, with some of them exhibiting pronounced antimicrobial, estrogenic, anticancer and other activities [1,2,3,4,5]. Research into the properties of stilbene derivatives began with the discovery of antibiotic activity against bacteria and fungi. These are demonstrated by natural stilbene derivatives from various plant species: grape, soybean, mulberry, cranberry, blueberry, and rhubarb [3,4]. An example is resveratrol which is naturally occurring in plants, and shows activity, against both gram-positive and gram-negative bacteria, with the range of antimicrobial activity being much broader against gram-positive bacteria. This is confirmed by numerous studies reported in the literature [6,7,8,9,10]. The differences can be explained by the fact that gram-negative bacteria are more complex in their structure and chemical composition [11]. The use of some antibiotics can lead to the development of multidrug-resistant organisms. The current standard in clinical practice is to use a combination of several antibiotics with different mechanisms of action to prevent the development of resistance and improve the outcome of therapy [12,13].

The Minimum Inhibitory Concentration (MIC) for gram-positive bacteria studied by Paulo et al. [11] was in the range of 50–200 µg/mL, while for gram-negative bacteria it was higher than 400 µg/mL. However, due to the poor solubility of resveratrol in water, concentrations higher than 400 µg/mL were not obtained. The stilbene derivatives discussed in this paper show a wide spectrum of biological activity, especially antimicrobial, fungistatic, fungitoxic and estrogenic action. Furthermore, they are characterized by high persistence and good solubility in water, which is why it seems very important to subject them to analytical studies and short- and long-term stability tests in aqueous solutions.

The purpose of this study was to summarize information on the analytical properties of stilbene derivatives, their biological activity, their possible applications as antiseptics, disinfectants and preservatives in various products, and to conduct research using environmental samples. Optimal conditions for the analysis of stilbene derivatives were developed using high-performance liquid chromatography and isotachophoresis.

## 2. Biological Activity of Stilbene Derivatives

One of the best-known stilbene derivatives is resveratrol. Resveratrol and its natural derivatives show only moderate antimicrobial activity. Nevertheless, the structure of resveratrol serves to further synthesize derivatives with antimicrobial, fungistatic, and fungicidal activity [14].

**Figure 1:**
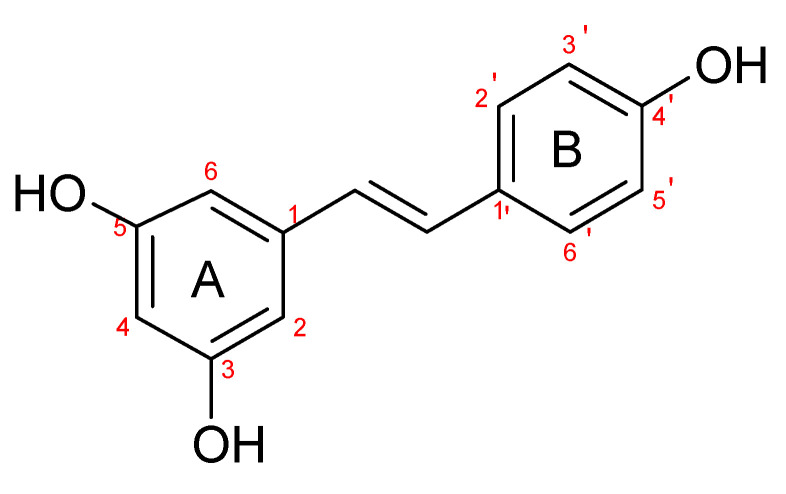
Chemical structure of resveratrol.

The presence of a hydroxyl group in the A ring of stilbene derivatives (2-hydroxy, 3-hydroxy, 4-hydroxy derivatives) is crucial to antimicrobial activity. If there is no hydroxyl substituent in the A ring, 2′,5′-dihydroxy substituents in the B ring are required for antimicrobial activity (Figure 1).

The effect of hydroxyl groups on antimicrobial activity does not seem surprising, as phenol is considered one of the primary antimicrobial agents [14]. Derivatives containing substituents (F, I, Br) show even greater antimicrobial activity. This fact is explained by a change in the partition coefficient and increased cell membrane permeability to fluoride derivatives, rather than as a direct effect of the presence of the substituents themselves [15,16].

*Trans*-resveratrol is a naturally occurring stilbene derivative showing antifungal activity against such fungi as *Pyricularia oryzae*, *Clodosporium cuccumerinum*, *Botrytis cinerea*, *Plasmopara viticola* and *Sphaeropsis sapinea* [17,18]. Examples of stilbene derivatives and their antimicrobial activity are shown in Table 1. During their antifungal activity, resveratrol and some of its analogs inhibit the enzyme tyrosinase found in fungi [19,20]. Some derivatives having methoxy groups in their structure show antifungal activity [14]. As a dimethyl derivative of resveratrol, pterostilbene has five times stronger antifungal properties in vitro than resveratrol. This indicates that methylation of hydroxyl groups may be important for the antifungal activity of phenolic derivatives. Such properties of methylated hydroxystilbene derivatives may be related to their greater ability to penetrate the lipophilic fungal cell membrane compared to the more hydrophilic resveratrol [21,22].

Stilbene derivatives can also enhance the effects of antibiotics [23,24]. The synergistic properties of stilbenes and antibiotics were first described by Kumar and co-workers in 2012 [5]. They tested two stilbene derivatives in vitro: *trans*-3,5,4′-trihydroxystilbene and 3,5-dihydroxy-4-isopropyl-*trans*-stilbene. The results of their study showed that when combined with ciprofloxacin or cefotaxime, they produced a synergistic pharmacological effect. In their study, they used the following bacterial strains: gram-positive strains—*Bacillus subtilis* MTCC 2756, *Staphylococcus aureus* MTCC 902, and gram-negative strains—*Escherichia coli* MTCC 2622 and *Pseudomonas aeruginosa* MTCC 2642. The combination of stilbene with an antibiotic resulted in a greater antibacterial effect than the sum of the actions of each individually, which offers the possibility of lowering the doses, thereby reducing toxic effects and delaying the development of bacterial resistance to a given antibiotic [5,24,25].

**Table 1:** Chemical structure and antimicrobial activity of selected stilbene derivatives.

Compound	Biological Activity	References
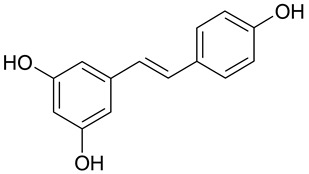	*P. oryzae*, *B. cinerea*, *P. capsici*, *P. viticola*, *C. cucumerinum*, *S. sapinea*, *P. colocasiae*, *C. gloeosporioides*	[26,27,28]
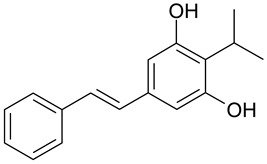	*C. albicans*, *F. Oxysporum*, *P. aphanidermatum*, *R. solani*, *E. turcicum*	[29,30]
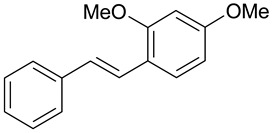	*B. cinerea*, *A. niger*, *B. subtilis*, *P. syringae*, *C. herbarum*	[27]
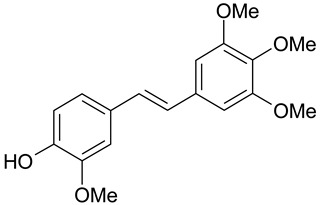	*P. viticola*, *B. cinerea*	[31]
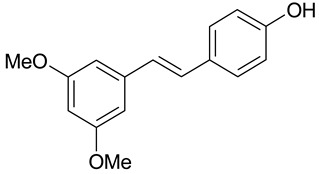	*C. albican*, *C. guilliermondii*, *C. famata*, *B. cinerea*, *P. viticola*	[32]
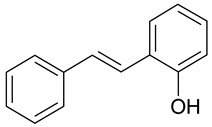	*A. niger*, *B. cinerea*, *C. herbarum*	[27]
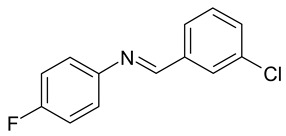	*A. niger*, *P. chrysogenum*	[33]
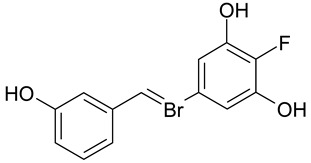	*B. brevis*, *B. subilis*, *M. luteus*, *M. miehei*	[34]
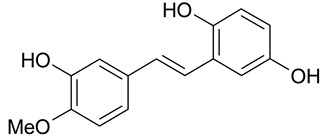	*B. brevis*, *B. subilis*, *M. luteus*	[34]
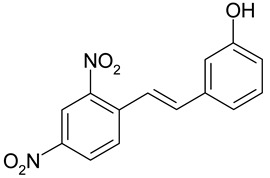	*E. coli*, *S. aureus*, *S. faecalis*, *B. subtilis*, *A. fumigatus*	[35]
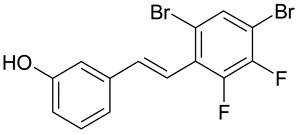	*R. miehei*, *P. notatum*, *C. graminicola*, *B. Cinerea*	[34]
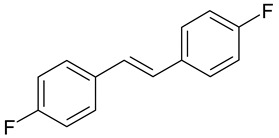	*A. niger*, *P. chrysogenum*	[36]
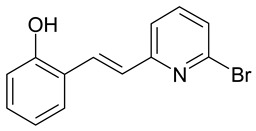	*M. indicus*	[37]
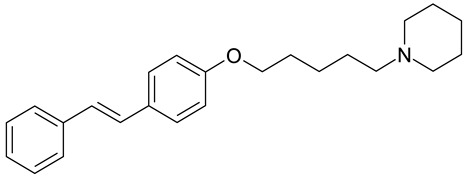	*S. aureus*, *S. faecalis*, *B. subtilis*, *A. fumigatus*	[38]
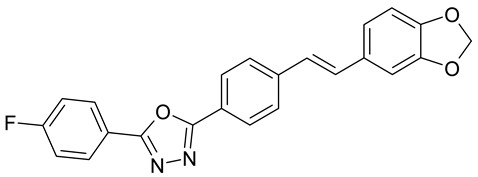	*B. cinerea*	[39]
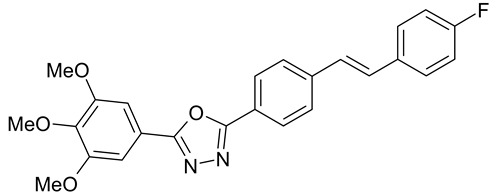	*C. lagenarium*	[40]

Before new drug combinations are administered to humans, they must be tested in vitro to determine their activity and possible toxicity. In the case of new stilbene derivatives, it is necessary to determine what interactions they are likely to undergo in combination with commonly used antibiotics before they can be used as antimicrobial agents. Therefore, further clinical studies in animals and humans are needed to conclusively determine whether combining these derivatives with other therapeutic substances will be safe. Although some interactions are predictable, clinical evidence of their existence is lacking. One of the derivatives is *trans*-3,5,4′-trihydroxystilbene, which shows antimicrobial activity against all bacteria tested, and synergism of action when combined with ciprofloxacin and cefotaxime. On the other hand, 3,5-dihydroxy-4-isopropyl-*trans*-stilbene is active only against gram-positive bacteria. In the case of the latter derivative, the synergism of action was observed in combination with ciprofloxacin, while the additive action was observed with cefotaxime [5]. The synergism of action of the aforementioned derivatives, i.e., *trans*-3,5,4′-trihydroxystilbene and 3,5-dihydroxy-4-isopropyl-*trans*-stilbene together with ciprofloxacin and cefotaxime may support the hypothesis that biologically active stilbene derivatives on an adjuvant (booster) basis may participate in antimicrobial therapy.

## 3. Estrogenic Effects

In recent years, much attention has been paid to compounds that, in addition to producing specific effects in the body and the ability to bioaccumulate, are capable of binding to estrogen receptors. Such compounds are called endocrinologically active hormone modulators or endocrine-disrupting compounds (EDCs) [21]. After entering the cell, sex hormones act by binding to specific intracellular receptors (ER, estrogen receptors *α* and *β*), which are also transcription factors. Upon binding to a ligand, the activated receptors reveal effects that enhance or silence the expression of target genes, thereby revealing their biological effects.

The effects of environmental estrogens primarily involve, but are not limited to, interaction with the estrogen receptor [41,42]. In trace amounts, phytoestrogens inhibit the activity of the enzyme (act as anti-estrogens), while in high concentrations, they act as typical estrogens [43]. One of the stilbenes belonging to the phytoestrogens is resveratrol. This compound competes with estradiol to bind to the human estrogen receptor [44,45,46]. Moreover, it activates the expression of estrogen-responsive reporter genes in many human cell lines. Resveratrol exhibits greater maximal transcriptional activity than estradiol, but this super agonism was not observed in all cell types. For example, resveratrol caused 2–4 times more activation of reporter plasmids than estradiol in MCF–7 breast cancer cells, but less in BG–1 ovarian cancer cells. These cell-type-specific effects of resveratrol resemble the well-known tissue- and species-specific effects exhibited by tamoxifen (a hormonal drug used to treat breast and endometrial cancer) [26]. They can act as estrogen receptor agonists in some tissues, such as the uterus, and as estrogen antagonists in the breast [12,43,44,45,46,47].

Neither stilbene nor stilbene oxide have estrogenic properties, and it is only through metabolism under the influence of liver microsomal enzymes that derivatives exhibiting potent effects are formed [13]. Sanoh et al. [26] showed that the liver microsomal enzyme system in the rat and human (CYP 1A1/2) metabolizes *trans*-stilbene to a 4-hydroxy derivative (*trans*-4-hydroxystilbene), which exhibits estrogenic effects. On the other hand, cis-stilbenes are not metabolically activated by the liver microsomal enzyme system to active hydroxyl derivatives and do not exhibit proestrogenic effects [13,21].

The hydroxyl group in the A-phenyl ring of stilbene is essential for the estrogenic effect [21,48]. The 4-nitro- and 4-amino- groups also contribute to this effect. The lipophilic group attached to the phenyl group is necessary for the maximum effect of stilbene derivatives, while the hydroxyl group at the 4′ position enhances the estrogenic effect of 4-hydroxy stilbene derivatives. Diethylstilbestrol (DES) and 4,4′-dihydroxy-α-methylstilbene exhibit a very strong estrogenic effect compared to 4,4′-dihydroxystilbene. This indicates that lipophilic substituents in the vinyl chain further enhance the estrogenic effects of stilbene derivatives. DES is the most popular stilbene derivative used in medicine for the treatment of prostate cancer, breast cancer and in pregnant women at risk of preterm labor. Compared to the hydroxyl derivatives of stilbene, resveratrol shows weaker estrogenic effects. This is probably due to the presence of two hydroxyl substituents at the 3′ and 5′ positions in the phenyl B ring [21].

## 4. Preservatives in Drugs and Problems in Their Selection

Preservative compounds originate from a group of antimicrobial substances and are used, among other things, in disinfection, antisepsis and in some cases of chemotherapy. In chemical terms, they belong to different groups and the range of their effects vary. The most commonly used compounds are phenols, alcohols, organic acids, biguanides, organic mercury compounds and quaternary ammonium bases [49]. At the concentrations used for drug preservation, these compounds perform basic tasks and mainly prevent the growth of or kill microorganisms [50,51]. The antimicrobial and antifungal effect of preservatives vary and depend on the concentration of a given preservative, its structure, pH of the solvent and the type of microorganism [52,53]. To date, no ideal chemical compound has been found that would fully meet all these requirements, as each of the compounds used has certain limitations [49,54].

The use of preservatives in drugs is regulated by law [55]. The requirements for parameters assessing the effectiveness of preservatives according to FP XII 2020 for parenteral and ocular preparations are shown in Table 2 [56]. Antimicrobial preservative properties are adequate if, under the test conditions after a specified time and at a specified temperature, there is a significant decrease or no increase in the number of viable microbial cells (cfu—colony-forming units) in the microbially contaminated test preparation after 28 days [56,57,58].

Test microorganism requirements, test conditions and performance criteria depend on the product category. Test organisms recommended by all pharmacopoeias (American, European, and Japanese) include: gram-positive granules, Staphylococcus aureus, gram-negative bacilli, Pseudomonas aeruginosa, fungi and molds, Aspergillus niger, yeast, Candida albicans [56,59,60]. In addition, the US Pharmacopoeia and the European Pharmacopoeia recommend the use of Escherichia coli strains. Preservatives can affect the natural bacterial flora found in the gastrointestinal tract [55]. In biological preparations, phenol still plays a major role as a preservative. However, it is used less and less frequently in the preservation of oral and topical preparations [58]. Bronopol, on the other hand, is not recommended in surface and ocular preparations due to concerns about the sensitizing effects of formaldehyde released under physiological conditions [61]. Preservatives from the alcohol group are generally considered safe. The exception is benzyl alcohol, which should not be present in preparations for low-birth-weight infants due to the risk of a fatal toxic shock syndrome [62,63]. Similarly, carboxylic acids (e.g., benzoic acid) can irritate the stomach, skin, eyes and mucous membranes [64,65].

The best-known metallic germicidal agents include copper and its alloys, as well as mercury compounds. Copper alloys have natural properties that kill a wide range of microorganisms [66,67]. Of the mercury compounds, organic compounds proved to be strongly bactericidal [68,69]. The list of the most commonly used preservatives for drug preservation is shown in Table 3.

The parabens listed in Table 3 as preservatives are currently very rarely used for drug preservation by many manufacturers due to ongoing research and debate over their harmfulness [70,71,72,73,74,75,76,77].

**Table 3:** List of preservatives.

Name of Preservative	Concentration [%]	Form of Drug
Thiomersal	0.005–0.01% [78] 0.0005–0.01% up to 0.007% [79]	vaccines, test solutions, topical preparations (creams), ear drops, eye drops
Parabens	0.065–0.15% up to 0.5%	eye medications ointments, oral medications
Benzalkonium chloride	up to 0.02% [80]	drugs and eye preparations,
Chlorhexidine	up to 0.05% [81]	eye medications

## 5. Stability and Analytics of Stilbene Derivatives

Given the biological activity of stilbenes and their derivatives and their wide range of applications, it is crucial to develop rapid and versatile methods for the analysis of these substances in samples with diverse and complex matrix composition. Each analytical procedure consists of several steps, and each of them is very important to keep the analytical errors as small as possible. There are more and less complex analytical procedures depending on the type of matrix and the nature of the chemicals to be analyzed. Therefore, only the correct determination of the conditions of analysis will determine their effectiveness. 

**Figure 2:**
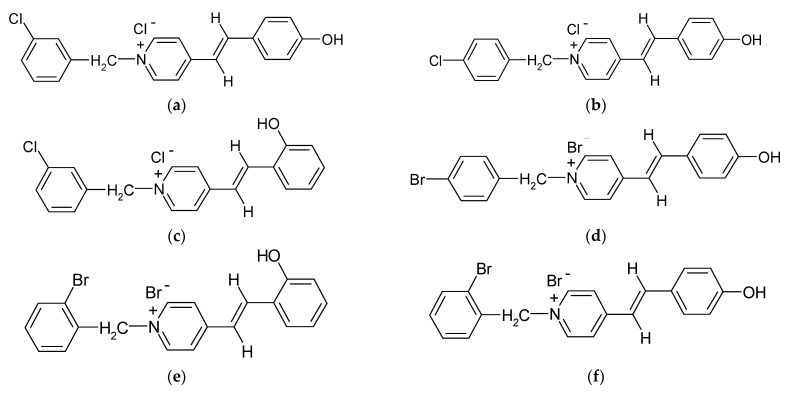
Chemical structures of stilbene derivatives: (**a**) (*E*)-1-(3-chlorobenzyl)-4-(4-hydroxystyryl)pyridin-1-ium chloride (**A1**); (**b**) (*E*)-1-(4-chlorobenzyl)-4-(4-hydroxystyryl)pyridin-1-ium chloride (**A2**); (**c**) (*E*)-1-(3-chlorobenzyl)-4-(2-hydroxystyryl)pyridin-1-ium chloride (**A3**); (**d**) (*E*)-1-(4-bromobenzyl)-4-(4-hydroxystyryl)pyridin-1-ium bromide (**A4**); (**e**) (*E*)-1-(2-bromobenzyl)-4-(2-hydroxystyryl)pyridin-1-ium bromide (**A5**); (**f**) (*E*)-1-(2-bromobenzyl)-4-(4-hydroxystyryl)pyridin-1-ium bromide (**A6**).

Aqueous solutions of the following compounds were used to determine the short- and long-term stability of the analyzed stilbene derivatives and to conduct tests using the isotachophoresis technique (Figure 2).

All the tested derivatives showed biological activity and were highly soluble in water [82,83,84,85]. Their antimicrobial properties and minimum inhibitory concentrations are shown in Table 4.

The most important physicochemical data for the stilbene derivatives in question are presented in Table 5.

### 5.1. Solid Phase Extraction

Sample preparation is the first and most important step in any analysis. It is also the most time-consuming process and takes about 70% of the total time required for any given analysis. For this reason, it is important to choose the most appropriate and suitable analytical technique at the very beginning of the analysis. One of the most commonly used methods to pre-concentrate a sample is solid-phase extraction (SPE, in various variants and scales). This method requires much less solvents and prevents analyte degradation to a greater extent than liquid–liquid extraction. In addition, SPE extraction allows better phase separation and proper recovery [84,85,86,87,88].

Depending on the needs, a wide range of different sorbents can be used in solid-phase extraction. Currently, the so-called dedicated phases are recommended for the extraction of a specific group of compounds. The most popular sorbents include reversed phases (C8 or C18) and the normal phase (aluminum oxide). Polymeric sorbents, graphitized carbon, as well as molecularly imprinted polymers can also be used. The choice of the right sorbent depends on its chemical and physical interaction with the selected analyte [89,90,91,92].

**Figure 3:**
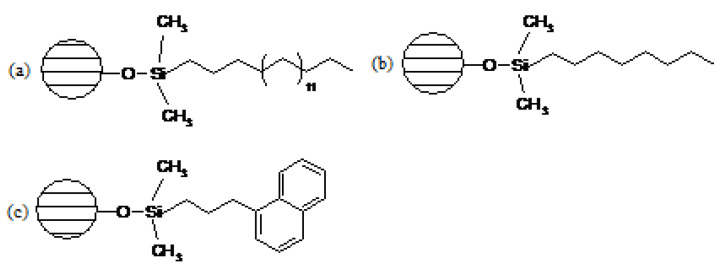
Structure of the stationary phase: (**a**) octadecyl, (**b**) octyl, (**c**) naphthylpropyl.

Three types of extraction columns were used to study the stability of stilbene derivatives: octyl, octadecyl and naphthylpropyl (Figure 3) [93,94,95,96,97]. It was shown that the naphthylpropyl-filled extraction column had the highest recoveries. Therefore, the process of extraction of the analyzed derivatives from individual samples was carried out using a naphthylpropyl phase column. The presence of an aryl group attached to the alkyl chain in the structure of the naphthylpropyl phase resulted in its greater activity in the isolation of analytes from various matrices due to additional π–π interactions. These interactions occur between isolated stilbene derivatives containing an aromatic ring and the terminal part of the ligand attached to the silica. In other cases of using octyl and octadecyl phases, only two types of active centers were present on the surface of the carrier, i.e., hydrophobic alkyl chains and residual silanols [98,99].

### 5.2. Short- and Long-Term Stability

The study to determine short- and long-term stability was conducted using three matrices, i.e., wastewater, surface water and distilled water. Due to possible changes over time, the content of the analyzed derivatives was determined at four time intervals, i.e., after 1 h, 7 days, 28 days and 12 months. Five samples of distilled water, surface water and wastewater were tested in each period. All the distilled water, surface water and wastewater solutions for extraction were stored in plastic bottles at 20–32 °C, depending on the season. Each sample was pre-filtered through a membrane strainer with a pore diameter of 3 µm to allow bacteria to pass into the solution. Stilbene derivatives were added to five samples of each type of water collected in parallel, yielding a concentration of 1000 µg/mL. SPE extraction was then carried out according to the procedure described [100]. The results for the short- and long-term stability of stilbene derivatives are shown in Table 6.

As indicated by the data presented, the highest average content of the analyzed stilbene derivatives was obtained in triple distilled water, and the lowest in wastewater. In the initial stage of the study, using the naphthylpropyl phase, the highest average content after 1 h was recorded for derivatives **A5** and **A6** from the matrix, which was distilled water. On the other hand, the lowest average content on this column was obtained for derivative **A1** for the most contaminated matrix, i.e., wastewater. Irrespective of the study period, the highest average content was recorded for derivative **A6** in distilled water, while the lowest was for derivatives **A1** and **A2** in wastewater. During the conducted analysis of stilbene derivatives, slight differences were found in the content decrease regardless of the type of matrix, both after 1 h, 7 days and after 28 days. A more pronounced decrease in the content of individual derivatives was found only after one year. The analyzed stilbene derivatives were hydrolytically stable, and resistant to oxygen and light, indicating that they could be used as preservatives. The preservative properties of the formulation are considered sufficient if no significant reduction in the preservative content is observed under the conditions of analysis after 28 days of residence in a microbially contaminated medium. In the described study, stilbene derivatives showed very good stability in the analyzed samples.

**Figure 4:**
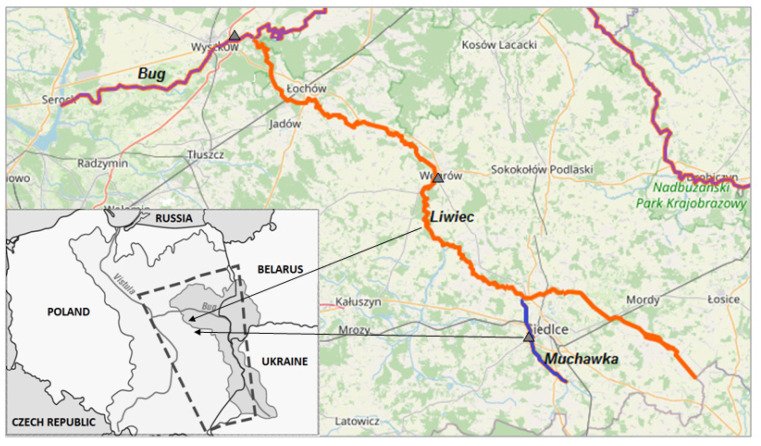
Map of surface water sampling for testing the stability of stilbene derivatives [85]. The purple line—the Bug River, the orange line—the Liwiec River, the blue line—the Muchawka River.

Another stability study was conducted using six stilbene derivatives and surface water collected from three different rivers. Twenty surface water samples each were collected in parallel from the Bug River in Wyszków, the Liwiec River in Węgrów, and the Muchawka River in Siedlce (Figure 4).

The Bug River is one of the longest rivers in Poland, flowing through Ukraine, Belarus and Poland. In recent years, some indicators of pollution of the studied rivers have improved, due in part to the construction of new wastewater treatment plants and sewage networks, as well as the modernization of the existing ones. Unfortunately, the waters of the Bug River still contain elevated levels of phosphorus and nitrogen, causing eutrophication, as well as sodium and potassium, contributing to the production of algal biomass. This primarily leads to the accumulation of blue-green algae, followed by the formation of blooms that reduce water transparency. In the border section, the Bug River is a receiver of untreated or insufficiently treated municipal wastewater from Ukraine and Belarus. The Bug River sampling site in Wyszkow is additionally polluted by municipal sewage. The city of Wyszkow discharges about 3000 m^3^ of sewage per day there, treated at a wastewater treatment plant with enhanced nutrient removal. In addition, two other rivers (Toczna and Cetynia) and the city of Sokolow Podlaski also discharge significant amounts of pollution into the Bug River. Total suspended solids are the main pollutant of this river [101,102,103,104,105,106]. The Liwiec River is the longest (over 142 km) left tributary of the Bug. The chemical status of its waters is described as moderate. This is due to the fact that the average and maximum concentrations of polycyclic aromatic hydrocarbons, including benzo [g,h,i]perylene and indeno [1,2,3-cd]pyrene, are exceeded [105]. The third river, the Muchawka, is a left tributary of the Liwiec, with a length of 32 km and a moderate water status. Thus, the condition of the water in these rivers is moderate and indicates the need for research to improve their quality.

The discussed stability studies of stilbene derivatives were carried out using a naphthylpropyl extraction column (Table 7). The highest results were obtained in samples collected from the Liwiec River and the Muchawka River, regardless of the time interval, while the lowest results were obtained for derivatives added to samples from the Bug River [106]. The obtained results showed good stability of the analyzed stilbene derivatives over the entire time interval studied, regardless of the type of matrix used for the study.

### 5.3. Isotachophoretic Separation

The isotachophoresis technique was used to determine the short- and long-term stability. The traditional technique used in preservative analysis is gas chromatography. However, it has some limitations. Problems occur in the analysis of polar substances with low volatility, as well as those with low thermal stability. Increasingly, mass spectrometry is being used for preservative analysis, which is more expensive and time consuming. High-performance liquid chromatography, on the other hand, uses various solvents that are most often organic and not necessarily environmentally friendly. For this reason, the isotachophoresis technique was used in the described studies. This technique has many advantages, such as short analysis time, the possibility of simultaneous determination of micro- and macro-components, uncomplicated sample preparation prior to analysis, and, above all, the use of non-toxic and biodegradable reagents and solvents. Isotachophoresis is classified as a “green chemistry” technique, while the precision and accuracy of the results obtained are better compared to traditional methods [107,108,109].

**Figure 5:**
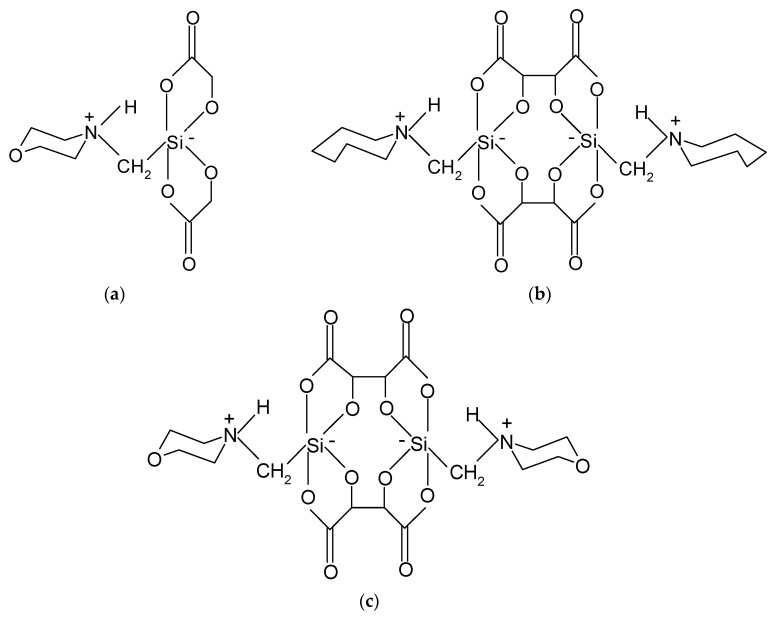
Structures of ES-silanate used as a terminal electrolyte: (**a**) 1-(*N*-morpholiniomethyl)spirobi(1-sila-2,5-dioxacyclopentan-3-on)at, (**b**) 4,4′-*bis*{1-(perhydroazepiniomethyl)[spirobi(1-sila-2,5-dioksacyklopentan-3-on)]at}, (**c**) 4,4′-*bis*[(1-morpholiniomethyl)spirobi(1-sila-2,5-dioxacyclopentan-3-on)at] [110,111].

In the studies using the isotachophoresis technique, aqueous solutions of three derivatives of electrostatically stabilized silanates (ES-silanates) were used as terminating electrolytes: 1-(N-morpholiniomethyl)spirobi(1-sila-2,5-dioxacyclopentan-3-one)at, 4,4′ -bis{1-(perhydroazepiniomethyl)[spirobi(1-sila-2,5-dioxacyclopentan-3-one)]at}, 4,4′-bis[(1-morpholiniomethyl)spirobi(1-sila-2,5-dioxacyclopentan-3-one)at] (Figure 5). The main factors in choosing these electrolytes were their documented non-toxicity to the environment and their biodegradability. In the previous studies, it was conclusively demonstrated that the ES–silanate derivatives in question can be counted among universal terminating electrolytes, as they can be used for both cation and anion analysis due to their zwitterionic molecular structure. In addition, these compounds are highly water soluble and hydrolytically stable and durable. The aforementioned electrolytes have been successfully used for many isotachophoretic analyses [110,111,112,113,114,115,116].

When using the isotachophoresis technique, the organic or inorganic ions to be analyzed migrate to the corresponding electrodes after applying an electric voltage. The use of two buffer systems of a leading electrolyte and a terminating electrolyte is necessary. The separation of a mixture of ions relies on the difference in their electrophoretic mobilities and in the mobilities of electrolytes used [117,118,119,120,121,122]. The ionic substances included in the leading electrolytes should have the highest possible mobility, while the terminating electrolytes should have the lowest mobility (μLd > μanalyte > μTm). The masses and sizes of ions, ionic charges and radii, the ability to form complexes or dissociate, changes in pH during the process or pK values, as well as the viscosities of solvents and their dielectric constant affect the differences in electrophoretic mobilities of individual ionic substances [123,124].

The terminating electrolytes used had significantly lower electrophoretic mobilities than the stilbene derivatives analyzed. At the same time, the stilbene derivatives showed very similar electrophoretic mobilities, which made their separation problematic. During the course of the experiments, the number of steps and analysis time, the value of current in each step, or the level of high voltage were changed on both the preseparation column and the analytical column. Separation using only a preseparation column was not possible due to small differences in the electrophoretic mobilities of the analyzed stilbene derivatives. The voltage value was changed from 9 kV to 15 kV, because no separation effect was obtained at a voltage lower than 9 kV. Finally, the optimal conditions for the separation and determination of six stilbene derivatives were developed in six steps of analysis (Table 8) [125].

**Figure 6:**
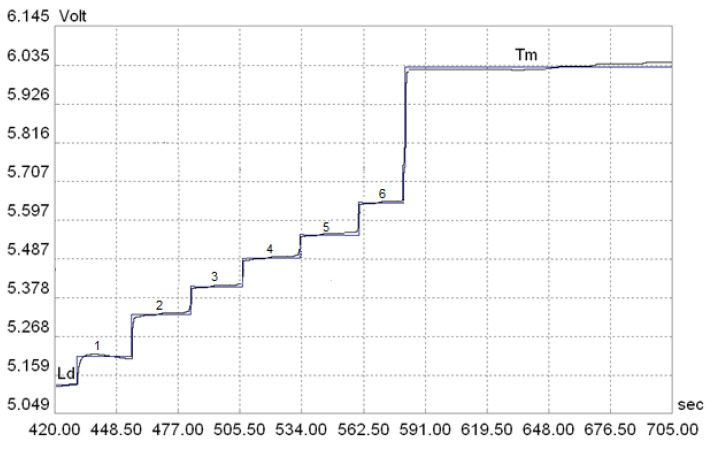
Isotachophoregram separation of the mixture of analyzed stilbene derivatives [125].

The zones of the individual components of the mixture were sharp and clearly separated from each other (Figure 6). The time of the isotachophoretic analysis of the stilbene derivatives did not exceed 12 min.

The isotachophoregrams obtained during the experiments, using different terminating electrolytes, showed only differences in the heights of the zones of these electrolytes. On the other hand, of the three terminating electrolytes used, the 4,4′-bis{1-(perhydroazepiniomethyl)[spirobi(1-sila-2,5-dioxacyclopentane-3-one)]at} derivative with the highest molecular weight was characterized by the lowest mobility. At the same time, it is worth noting that very good separation of the analyzed stilbene derivatives was obtained for each of the terminating electrolytes used.

## 6. Conclusions

Stability studies expand the potential applications of biologically active stilbene derivatives and advance the understanding of this group of compounds. The derivatives described in this work were characterized by good short- and long-term stability in various environmental matrices. The development of new application possibilities for stilbene derivatives expands the list of available substances with fungistatic and fungitoxic properties with the possibility of using them in various industries and in environmental protection. The advantage of using these derivatives may also be the fact that they do not have negative effects on human health and the environment. In addition, the antibacterial and antifungal activity of such compounds, as well as the possibility of using them as preservatives, are new areas of application for this class of compounds.

The use of the isotachophoresis technique, which is categorized as a green chemistry technique, enabled the development of optimal conditions for the qualitative and quantitative analysis of stilbene derivatives. Particular attention was paid to the use of non-toxic and biodegradable terminating electrolytes, belonging to electrostatically stabilized silanates.

## Figures and Tables

**Table 2:** Effective performance criteria for preservatives according to FP XII.

Microorganisms	Criteria	Log Reduction				
		6 h	24 h	7 Days	14 Days	28 Days
Bacteria	A	2	3	-	-	BW
	B	-	1	3	-	BN
Fungi	A	-	-	2	-	BN
	B	-	-	-	1	BN

BW—no growth of viable microorganisms, BN—no increase in the number of microorganisms.

**Table 4:** Antimicrobial properties of stilbene derivatives [70].

Compound	Minimum Inhibitory Concentration, μg/mL								
	1	2	3	4	5	6	7	8	9
(**A1**)	100	500	500	100	1000	1000	>500	>500	>500
(**A2**)	100	100	500	100	500	1000	>500	>500	>500
(**A3**)	7.5	100	100	100	1000	1000	>500	>500	>500
(**A4**)	5	500	500	100	1000	1000	>500	>500	>500
(**A5**)	100	500	500	500	1000	1000	>500	>500	>500
(**A6**)	7.5	500	100	100	1000	1000	>500	>500	>500

**1**—Staphylococcus aureus 209P FDA, **2**—Streptococcus faecalis ATCC 8040, **3**—Bacillus subtilis ATCC 1633, **4**—Escherichia coli PZHO 26B6, **5**—Klebsiella pneumoniae 231, **6**—Pseudomonas aeruginosa 5 R1, **7**–Candida albicanus PCM 1409 PZH, **8**—Microsporum gypseum K1, **9**—Aspergillus fumigatus C1.

**Table 5:** Basic chemical and physical data of stilbene derivatives.

Compound	m.p. °C	IR(KBr) (cm^−1^) δ_CH=CH_	^1^H NMR δ (ppm) DMSO–*d*_6_ CH_2_–N
(**A1**)	227–230	970	5.81 s
(**A2**)	240–243	985	5.81 s
(**A3**)	209–212	965	5.82 s
(**A4**)	256–259	995	5.88 s
(**A5**)	218–221	980	5.90 s
(**A6**)	247–249	955	5.86 s

**Table 6:** Mean content values of the analyzed stilbene derivatives in distilled, surface and wastewater samples after 1 h, 7 days, 28 days and 12 months (SD ≤ 5%) [100].

Time	Matrix	Average Value, µg/mL					
		A1	A2	A3	A4	A5	A6
1 h	distilled water 3x	893.8	881.9	903.1	886.4	921.9	917.4
	surface water	888.1	883.4	887.9	882.7	918.0	913.6
	wastewater	871.9	876.4	879.1	878.3	913.4	909.0
7 days	distilled water 3x	884.5	887.1	881.7	882.6	911.9	903.5
	surface water	873.1	870.2	863.8	874.5	902.5	875.1
	wastewater	860.7	864.8	861.0	860.1	864.9	860.6
28 days	distilled water 3x	795.2	788.1	794.1	786.9	790.0	807.6
	surface water	789.1	787.5	786.5	791.0	795.8	781.5
	wastewater	781.6	780.3	780.2	783.3	777.8	763.5
1 year	distilled water 3x	715.3	703.7	705.0	713.4	695.1	721.2
	surface water	690.1	687.8	681.1	678.9	679.3	645.5
	wastewater	686.2	679.7	696.8	686.7	701.2	680.7

**Table 7:** Mean content values for (*E*)-1-(3-chlorobenzyl)-4-(4-hydroxystyryl)pyridin-1-ium chloride (**A1**), (*E*)-1-(4-chlorobenzyl)-4-(4-hydroxystyryl)pyridin-1-ium chloride (**A2**), (*E*)-1-(3-chlorobenzyl)-4-(2-hydroxystyryl)pyridin-1-ium chloride (**A3**), (*E*)-1-(4-bromobenzyl)-4-(4-hydroxystyryl)pyridin-1-ium bromide (**A4**), (*E*)-1-(2-bromobenzyl)-4-(2-hydroxystyryl)pyridin-1-ium bromide (**A5**), (*E*)-1-(2-bromobenzyl)-4-(4-hydroxystyryl)pyridin-1-ium bromide (**A6**), in the surface water samples after 1 h, 7 days, 28 days and 12 months, obtained in the naphthylpropyl column [106].

Times	Water from the River	Average Value (µg/mL), SD (%)					
		(A1)	(A2)	(A3)	(A4)	(A5)	(A6)
1 h	Liwiec	893.1 ± 4.5	887.2 ± 3.9	829.9 ± 4.0	837.4 ± 3.6	877.2 ± 4.9	849.1 ± 4.6
	Muchawka	867.3 ± 4.1	882.7 ± 3.5	858.6 ± 4.2	823.6 ± 4.6	865.7 ± 5.5	864.6 ± 5.3
	Bug	779.7 ± 3.4	773.3 ± 3.5	863.4 ± 4.1	859.1 ± 4.8	783.8 ± 3.9	854.4 ± 4.6
7 days	Liwiec	783.7 ± 3.2	782.3 ± 4.3	780.9 ± 3.5	768.5 ± 3.6	787.3 ± 4.4	786.1 ± 3.9
	Muchawka	767.5 ± 4.2	764.7 ± 4.1	752.1 ± 4.5	775.1 ± 4.0	774.2 ± 5.2	749.1 ± 3.6
	Bug	681.7 ± 3.6	683.4 ± 4.1	694.9 ± 3.7	700.6 ± 3.9	693.6 ± 4.7	688.9 ± 4.7
28 days	Liwiec	591.1 ± 4.4	596.9 ± 4.1	590.3 ± 4.3	557.6 ± 4.4	587.9 ± 4.3	593.3 ± 5.1
	Muchawka	566.5 ± 3.9	591.6 ± 3.4	595.4 ± 4.5	583.5 ± 4.1	581.5 ± 4.4	590.1 ± 4.0
	Bug	570.2 ± 4.9	587.3 ± 4.2	571.8 ± 4.4	563.9 ± 4.0	567.3 ± 5.5	567.8 ± 4.7
12 months	Liwiec	295.2 ± 3.7	283.1 ± 3.2	295.4 ± 4.4	321.2 ± 3.5	293.3 ± 4.1	301.4 ± 4.8
	Muchawka	281.6 ± 3.8	268.9 ± 3.6	279.7 ± 3.4	345.5 ± 4.7	277.9 ± 5.2	288.7 ± 3.9
	Bug	276.8 ± 3.6	286.8 ± 4.2	301.3 ± 4.6	297.7 ± 3.7	276.4 ± 3.9	299.3 ± 5.1

**Table 8:** Best conditions for isotachophoretic separation of a mixture of analyzed stilbene derivatives. High voltage limit of 12 kV, sample rate of 50 smp/s, polarity—cations. Ld-1: 3 × 10^−3^ mol/L *Bis*-*Tris* Propane (BTP), 1.5 × 10^−3^ mol/L *β*-alanine, 0.1% HEC, HCl (final pH = 3.8). Ld-2: 1.5 × 10^−3^ mol/L *β*-alanine, 0.1% HEC, HCl (final pH = 3.8). Terminating electrolyte (Tm): 10^−3^ mol/L 4,4′-*bis*{1-(perhydroazepiniomethyl)[spirobi(1-sila-2,5-dioksacyklopentan-3-on)]ate} [125].

Considered Parameters					
Stage	Time [s]	Intensity [µA]	Comp [10 mV]	Column	Conductometric Detector
1	100	100	0	top	
2	250	250	0	top	X
3	65	10	0	lower	
4	10	95	0	lower	
5	30	75	50	lower	
6	250	40	0	lower	X

## Data Availability

Not applicable.

## Sample Availability

Samples of the compounds (stilbenes and ES-silanates) are available from the authors.

## References

[1] Pecyna P., Wargula J., Murias M., Kucinska M. (2020). More than Resveratrol: New Insights into Stilbene-Based Compounds. Biomolecules.

[2] Valletta A., Iozia L.M., Leonelli F. (2021). Impact of Environmental Factors on Stilbene Biosynthesis. Plants.

[3] Xia N., Daiber A., Förstermann U., Li H. (2017). Antioxidant effects of resveratrol in the cardiovascular system. Br. J. Pharmacol..

[4] Olas B. (2003). Antioxidants present in diet as anti atherosclerosis factors. Kosmos Probl. Nauk Biol..

[5] Kumar S.N., Siji J.V., Nambisan B., Mohandas C. (2012). Activity and synergistic interactions of stilbenes and antibiotic combinations against bacteria in vitro. World J. Microbiol. Biotechnol..

[6] Mahady G.B., Pendland S.L. (2000). Resveratrol Inhibits The Growth of Helicobacter Pylori in Vitro. Am. J. Gastroenterol..

[7] Chan M.M.-Y. (2002). Antimicrobial effect of resveratrol on dermatophytes and bacterial pathogens of the skin. Biochem. Pharmacol..

[8] Shan B., Cai Y.-Z., Brooks J.D., Corke H. (2008). Antibacterial properties of Polygonum cuspidatum roots and their major bioactive constituents. Food Chem..

[9] Wang W.-B., Lai H.-C., Hsueh P.-R., Chiou R.Y.-Y., Lin S.-B., Liaw S.-J. (2006). Inhibition of swarming and virulence factor expression in Proteus mirabilis by resveratrol. J. Med. Microbiol..

[10] Docherty J.J., Fu M.M., Tsai M. (2001). Resveratrol selectively inhibits Neisseria gonorrhoeae and Neisseria meningitidis. J. Antimicrob. Chemother..

[11] Paulo L., Ferreira S., Gallardo E., Queiroz J., Domingues F. (2010). Antimicrobial activity and effects of resveratrol on human pathogenic bacteria. World J. Microbiol. Biotechnol..

[12] Almstrup K., Fernández M.F., Petersen J.H., Olea N., Skakkebaek N.E., Leffers H. (2002). Dual effects of phytoestrogens result in u-shaped dose-response curves. Environ. Health Perspect..

[13] Gehm B.D., McAndrews J.M., Chien P.-Y., Jameson J.L. (1997). Resveratrol, a polyphenolic compound found in grapes and wine, is an agonist for the estrogen receptor. Proc. Natl. Acad. Sci. USA.

[14] Albert S., Horbach R., Deising H.B., Siewert B., Csuk R. (2011). Synthesis and antimicrobial activity of (E) stilbene derivatives. Bioorganic Med. Chem..

[15] Jeandet P., Douillet-Breuil A.-C., Bessis R., Debord S., Sbaghi M., Adrian M. (2002). Phytoalexins from the Vitaceae: Biosynthesis, Phytoalexin Gene Expression in Transgenic Plants, Antifungal Activity, and Metabolism. J. Agric. Food Chem..

[16] Aslam S.N., Stevenson P.C., Kokubun T., Hall D.R. (2009). Antibacterial and antifungal activity of cicerfuran and related 2-arylbenzofurans and stilbenes. Microbiol. Res..

[17] Weber K., Schulz B., Ruhnke M. (2011). Resveratrol and its antifungal activity against Candida species. Mycoses.

[18] Houillé B., Papon N., Boudesocque L., Bourdeaud E., Besseau S., Courdavault V., Enguehard-Gueiffier C., Delanoue G., Guérin L., Bouchara J.-P. (2014). Antifungal Activity of Resveratrol Derivatives against Candida Species. J. Nat. Prod..

[19] Kato E., Tokunaga Y., Sakan F. (2009). Stilbenoids Isolated from the Seeds of Melinjo (*Gnetum gnemon* L.) and Their Biological Activity. J. Agric. Food Chem..

[20] Shina N.H., Ryu S.Y., Choi E.J., Kangc S.H., Changd I.L.M., Min K.R., Kim Y. (1998). Oxyresveratrol as the Potent Inhibitor on Dopa Oxidase Activity of Mushroom Tyrosinase. Biochem. Biophys. Res. Commun..

[21] Sanoh S., Kitamura S., Sugihara K., Fujimoto N., Ohta S. (2003). Estrogenic Activity of Stilbene Derivatives. J. Health Sci..

[22] Singh D., Chauhan N., Koli M., Nayak S.K., Subramanian M. (2022). Dimer stilbene, a resveratrol analogue exhibits synergy with antibiotics that target protein synthesis in eradicating Staphylococcus aureus infection. Biochimie.

[23] Cebrián R., Li Q., Peñalver P., Belmonte-Reche E., Andrés-Bilbao M., Lucas R., de Paz M.V., Kuipers O.P., Morales J.C. (2022). Chemically Tuning Resveratrol for the Effective Killing of Gram-Positive Pathogens. J. Nat. Prod..

[24] Chong J., Poutaraud A., Hugueney P. (2009). Metabolism and roles of stilbenes in plants. Plant Sci..

[25] Adwan G., Mhanna M. (2008). Synergistic Effects of plant extracts and antibiotics on Staphylococcus aureus strains isolated from clinical specimens. Middle-East J. Sci. Res..

[26] Adrian M., Jeandet P. (2012). Effects of resveratrol on the ultrastructure of Botrytis cinerea conidia and biological significance in plant/pathogen interactions. Fitoterapia.

[27] Bostanghadiri N., Pormohammad A., Chirani A.S., Pouriran R., Erfanimanesh S., Hashemi A. (2017). Comprehensive review on the antimicrobial potency of the plant polyphenol Resveratrol. Biomed. Pharmacother..

[28] Kumar S.N., Nambisan B. (2014). Antifungal Activity of Diketopiperazines and Stilbenes Against Plant Pathogenic Fungi In Vitro. Appl. Biochem. Biotechnol..

[29] Park H.B., Crawford J.M. (2015). Lumiquinone A, an α-Aminomalonate-Derived Aminobenzoquinone from *Photorhabdus luminescens*. J. Nat. Prod..

[30] Shi D., An R., Zhang W., Zhang G., Yu Z. (2017). Stilbene Derivatives from *Photorhabdus temperata* SN259 and Their Antifungal Activities against Phytopathogenic Fungi. J. Agric. Food Chem..

[31] Chalal M., Klinguer A., Echairi A., Meunier P., Vervandier-Fasseur D., Adrian M. (2014). Antimicrobial Activity of Resveratrol Analogues. Molecules.

[32] Li D.-D., Zhao L.-X., Mylonakis E., Hu G.-H., Zou Y., Huang T.-K., Yan L., Wang Y., Jiang Y.-Y. (2014). *In Vitro* and *In Vivo* Activities of Pterostilbene against Candida albicans Biofilms. Antimicrob. Agents Chemother..

[33] Karki S.S., Butle S.R., Shaikh R.M., Zubaidha P.K., Pedgaonkar G.S., Shendarkar G.S., Rajput C.G. (2010). Synthesis and biological evaluation of some novel substituted N-benzylideneaniline derivatives. Res. J. Pharm. Biol. Chem. Sci..

[34] Bavaresco L., Mattivi F., De Rosso M., Flamini R. (2012). Effects of elicitors, viticultural factors, and enological practices on resveratrol and stilbenes in grapevine and wine. Mini-Rev. Med. Chem..

[35] Wyrzykiewicz E., Błaszczak A., Kędzia B. (2000). Synthesis and antimicrobial activity of (E)-acetoxystilbenes and a,a′-dibromoacetoxybibenzyls. Il Farm..

[36] Karki S.S., Bhutle S.R., Pedgaonkar G.S., Zubaidha P.K., Shaikh R.M., Rajput C.G., Shendarkar G.S. (2011). Synthesis and biological evaluation of some stilbene-based analogues. Med. Chem. Res..

[37] Chandrasekara Reddy G., Shiva Prakash S., Diwakar L. (2015). Stilbene heterocycles: Synthesis, antimicrobial, antioxidant and anti-cancer activities. Pharm. Innov..

[38] Wyrzykiewicz E., Wendzonka M., Kędzia B. (2006). Synthesis and antimicrobial activity of new (E)-4-[piperidino (4′-methylpiperidino-, morpholino-) N-alkoxy]stilbenes. Eur. J. Med. Chem..

[39] Jian W., He D., Song S. (2016). Synthesis, Biological Evaluation and Molecular Modeling Studies of New Oxadiazole-Stilbene Hybrids against Phytopathogenic Fungi. Sci. Rep..

[40] Jian W., He D., Xi P., Li X. (2015). Synthesis and Biological Evaluation of Novel Fluorine-Containing Stilbene Derivatives as Fungicidal Agents against Phytopathogenic Fungi. J. Agric. Food Chem..

[41] Piver B., Berthou F., Dreano Y., Lucas D. (2001). Inhibition of CYP3A, CYP1A and CYP2E1 activities by resveratrol and other non volatile red wine components. Toxicol. Lett..

[42] Wierzchowski M., Dutkiewicz Z., Gielara-Korzańska A., Korzański A., Teubert A., Teżyk A., Stefański T., Baer-Dubowska W., Mikstacka R. (2017). Synthesis, biological evaluation and docking studies of trans -stilbene methylthio derivatives as cytochromes P450 family 1 inhibitors. Chem. Biol. Drug Des..

[43] Kobylka P., Kucinska M., Kujawski J., Lazewski D., Wierzchowski M., Murias M. (2022). Resveratrol Analogues as Selective Estrogen Signaling Pathway Modulators: Structure–Activity Relationship. Molecules.

[44] Lephart E.D. (2021). Phytoestrogens (Resveratrol and Equol) for Estrogen-Deficient Skin—Controversies/Misinformation versus Anti-Aging In Vitro and Clinical Evidence via Nutraceutical-Cosmetics. Int. J. Mol. Sci..

[45] Shah A.A., Shah A., Kumar A., Lakra A., Singh D., Nayak Y. (2022). Phytoestrogenic Potential of Resveratrol by Selective Activation of Estrogen Receptor-α in Osteoblast Cells. Rev. Bras. Farm..

[46] Rimando A.M., Suh N. (2008). Biological/Chemopreventive Activity of Stilbenes and their Effect on Colon Cancer. Planta Med..

[47] Kuršvietienė L., Stanevičienė I., Mongirdienė A., Bernatonienė J. (2016). Multiplicity of effects and health benefits of resveratrol. Medicina.

[48] Fang H., Tong W., Shi L.M., Blair R., Perkins R., Branham W., Hass B.S., Xie Q., Dial S.L., Moland C.L. (2001). Structure−Activity Relationships for a Large Diverse Set of Natural, Synthetic, and Environmental Estrogens. Chem. Res. Toxicol..

[49] Elder D.P. (2012). Effective formulation development strategies for poorly soluble active pharmaceutical ingredients (APIs). Am. Pharm. Rev..

[50] Stanojevic D., Comic L.J., Stefanovic O., Solujic-Sukdolak S. (2009). Antimicrobial effects of sodium benzoate, sodium nitrite and potassium sorbate and their synergistic action in vitro. Bulg. J. Aqric. Sci..

[51] Bojarowicz H., Fronczak P., Krysiński J. (2018). Can cosmetics be preservative-free?. Hygeia Public Health.

[52] Elder D.P., Crowley P.J. (2012). Antimicrobial Preservatives Part Three: Chalenges Facing Preservative Systems. www.americanpharmaceutical-review.com.

[53] Strilets O.P., Petrovska L.S., Baranova I.I., Bespala Y.O. (2017). A study of antimicrobial activity of foam-washing agent specimens at acidic pH values. Ann. Mechnikov’s Institude.

[54] Hall M.J., Middleton R.F., Westmacott D. (1983). The fractional inhibitory concentration (FIC) index as a measure of synergy. J. Anti-microb. Chem..

[55] Regulation of the Minister of Health on Preservatives, Sweeteners, Colors and Antioxidants That May Be Included in the Com-Position of Medicinal Products. Dz.U. 2003 nr 19, poz. 169. https://isap.sejm.gov.pl/isap.nsf/DocDetails.xsp?id=WDU20030190169.

[56] (2022). Pharmacopeia Poland.

[57] Penha F.M., Rodrigues E.B., Maia M., Furlani B.A., Regatieri C., Melo G.B., Magalhães J.O., Manzano R., Farah M.E. (2010). Retinal and Ocular Toxicity in Ocular Application of Drugs and Chemicals—Part II: Retinal Toxicity of Current and New Drugs. Ophthalmic Res..

[58] Walsh F. (2005). Muslim Fear Hamper Drive to Eradicate Polio. The Observer.

[59] United States Pharmacopeia, General Chapter Antimicrobial Effectiveness Testing; USP 34–NF29; United States Pharmacopeial Convention: Rockville, MD, USA, 1 January 2015. www.amazon.com/United-States-Pharmacopeia-National-Formulary/dp/1936424320.

[60] European Directorate for Quality of Medicines (2020). European Pharmacopeia 5.1.3.

[61] Kireche M., Peiffer J.-L., Antonios D., Fabre I., Giménez-Arnau E., Pallardy M., Lepoittevin J.-P., Ourlin J.-C. (2011). Evidence for Chemical and Cellular Reactivities of the Formaldehyde Releaser Bronopol, Independent of Formaldehyde Release. Chem. Res. Toxicol..

[62] Anon (1982). Benzyl alcohol may be toxic to newborns. FDA Drug. Bull..

[63] Cahill E., Rowe R.C., Sheskey P.J., Weller P.J. (2006). Benzyl Alcohol Monograph. Handbook of Pharmaceutical Excipients.

[64] Weller P.J., Rowe R.C., Sheskey P.J., Weller P.J. (2007). Benzoic Acid Monigraph. Handbook of Pharmaceutical Excipients.

[65] Downard C.D., Roberts L.J., Morrow J.D. (1995). Topical benzoic acid induces the increased biosynthesis of prostaglandin D2 in human skin in vivo*. Clin. Pharmacol. Ther..

[66] Abraham J., Dowling K., Florentine S. (2021). Can copper products and surfaces reduce the spread of infectious microorganisms and hospital-acquired infections?. Materials.

[67] Różańska A., Chmielarczyk A., Romaniszyn D., Sroka-Oleksiak A., Bulanda M., Walkowicz M., Osuch P., Knych T. (2017). Antimicrobial Properties of Selected Copper Alloys on Staphylococcus aureus and Escherichia coli in Different Simulations of Environmental Conditions: With vs. without Organic Contamination. Int. J. Environ. Res. Public Health.

[68] Barregard L., Rekić D., Horvat M., Elmberg L., Lundh T., Zachrisson O. (2011). Toxicokinetics of Mercury after Long-Term Repeated Exposure to Thimerosal-Containing Vaccine. Toxicol. Sci..

[69] Aronson J.K. (2015). Meyler’s Side Effects of Drugs.

[70] Esposito S., Principi N., Cornaglia G. (2014). Barriers to the vaccination of children and adolescents and possible solutions. Clin. Microbiol. Infect..

[71] Geier D.A., Geier M.R. (2006). An assessment of downward trends in neurodevelopmental disorders in the United States following removal of Thimerosal from childhood vaccines. Experiment.

[72] Tan M., Parkin J. (2000). Route of decomposition of thiomersal (thimerosal). Int. J. Pharm..

[73] Trümpler S., Meermann B., Nowak S., Buscher W., Karst U., Sperling M. (2014). In vitro study of thimerosal reactions in human whole blood and plasma surrogate samples. J. Trace Elem. Med. Biol..

[74] Geier D.A., Kern J.K., Geier M.R. (2018). Premature Puberty and Thimerosal-Containing Hepatitis B Vaccination: A Case-Control Study in the Vaccine Safety Datalink. Toxics.

[75] Vena G.A., Foti C., Grandolfo M., Angelini G. (1994). Mercury exanthem. Contact Dermat..

[76] Lohiya G. (1987). Asthma and urticaria after hepatitis B vaccination. West. J. Med..

[77] Aberer W. (1991). Vaccination despite thimerosal sensitivity. Contact Dermat..

[78] Kern J.K., Haley B.E., Geier D.A., Sykes L.K., King P.G., Geier M.R. (2013). Thimerosal Exposure and the Role of Sulfation Chemistry and Thiol Availability in Autism. Int. J. Environ. Res. Public Health.

[79] Podgórska A., Puścion-Jakubik A., Grodzka A., Naliwajko S.K., Markiewicz-Zukowska R., Socha K. (2021). Natural and conven-tional cosmetics-mercury exposure assessment. Molecules.

[80] Liebert M.A. (1985). Final report on the safety assessment of benzethonium chloride and methylbenzethonium chloride. J. Am. Coll. Toxicol..

[81] Rove R.C., Sheskey P.J., Owen S.C. (2006). Handbook of Pharmaceutical Excipients.

[82] Prukala W., Wyrzykiewicz E., Kedzia B. (1995). Synthesis and antimicrobial properties of N-substituted halides of (E)-azastilbenols. Il Farm..

[83] Prukała D., Prukała W., Małkiewicz K., Szymalska M., Witkowska-Krajewska E., Kluska M. (2010). New methodology of separation and determination of biologically active isomers of nitrobenzyl azastilbene derivatives. J. Liq. Chromatogr. Relat. Technol..

[84] Al-Suod H., Ratiu I.-A., Gadzała-Kopciuch R., Górecki R., Buszewski B. (2023). Identification and quantification of cyclitols and sugars isolated from different morphological parts of *Raphanus sativus* L.. Nat. Prod. Res..

[85] Mencin M., Mikulic-Petkovsek M., Veberic R., Terpinc P. (2021). Development and optimisation of solid-phase extraction of ex-tractable and bound phenolic acids in spelt (*Triticum spelta* L.) seeds. Antioxidants.

[86] Nawała J., Szala M., Dziedzic D., Gordon D., Dawidziuk B., Fabisiak J., Popiel S. (2020). Analysis of samples of explosives excavated from the Baltic Sea floor. Sci. Total Environ..

[87] Wianowska D. (2022). Combination of sea sand disruption method and ion-pair solid-phase extraction for effective isolation and purification of chlorogenic acid from plants prior to the HPLC determination. Molecules.

[88] Cigalski P., Kosobucki P. (2020). Recent Materials Developed for Dispersive Solid Phase Extraction. Molecules.

[89] Awang M.A., Chua L.S., Abdullah L.C. (2022). Solid-Phase Extraction and Characterization of Quercetrin-Rich Fraction from *Melastoma malabathricum* Leaves. Separations.

[90] Kluska M., Krajewska E., Jabłońska J., Prukała W. (2019). New Applications and Analysis of (E)-Azastilbenes in Environmental Samples. Crit. Rev. Anal. Chem..

[91] Buszewski B., Szultka-Młyńska M. (2012). Past, Present, and Future of Solid Phase Extraction: A Review. Crit. Rev. Anal. Chem..

[92] Jabłońska J., Kluska M., Erchak N. (2020). Development of a procedure for the isolation of electrostatically stabilized silanates from wheat samples. Przem. Chem..

[93] Kluska M., Liepinsh E., Pypowski K., Erchak N. (2008). Separation of Azepinio-Methyl Derivatives of ES-Silanates by the Use of Aryl Stationary Phases in HPLC. J. Liq. Chromatogr. Relat. Technol..

[94] Kluska M., Liepinsh E., Pypowski K., Chrząścik I., Michel M., Erchak N. (2008). Separation of Selected Derivatives of Hoszczawa-Silanates, Taking Advantage of π–π Interactions. J. Liq. Chromatogr. Relat. Technol..

[95] Prukała W., Prukała D., Pypowski K., Chrząścik I., Kluska M. (2008). Chromatography of Biologically Active Chlorides of (*E*)-N-*o*-(*m*- or *p*-)chlorobenzyl-γ-azastilbenols-2′(3′ or 4′). J. Liq. Chromatogr. Relat. Technol..

[96] Prukała W., Pypowski K., Chrząścik I., Kluska M. (2008). Separation of Biologically Active Isomers of (E)-N-Meta- and Para-Nitroazastilbenes by the HPLC Technique. J. Liq. Chromatogr. Relat. Technol..

[97] Gadzała-Kopciuch R., Kluska M., Wełniak M., Buszewski B. (2005). Silicon dioxide surfaces with aryl interaction sites for chromatographic applications. Mater. Chem. Phys..

[98] Kluska M., Jabłońska J., Erchak N. (2021). Analytics and Application of Biologically Active Pentacoordinate Electrostatically Stabilized Silanates. Crit. Rev. Anal. Chem..

[99] Jabłońska J., Kluska M., Erchak N., Popiel S. (2020). Development of an extraction procedure and analysis of electrostatically stabilized silanates from aqueous solutions. Oceanol. Hydrobiol. Stud..

[100] Witkowska-Krajewska E., Kluska M., Prukała W., Mikulewicz M., Chojnacka K., Małkiewicz K. (2018). Study on stability of (E)-azastilbenes as disinfectants and preservatives as well as their recovery from aqueous solutions. Przem. Chem..

[101] Gopchak I., Kalko A., Basiuk T., Pinchuk O., Gerasimov I., Yaromenko O., Shkirynets V. (2020). Assessment of surface water pol-lution in Western Bug River within the cross-border section of Ukraine. J. Water Land Dev..

[102] Gopchak I., Basiuk T., Bialyk I., Pinchuk O., Gerasimov I. (2019). Dynamics of changes in surface water quality indicators of the Western Bug River basin within Ukraine using GIS technologies. J. Water Land Dev..

[103] Hagemann N., Blumensaat F., Tavares F., Trumper J., Burmeister C., Moynihan R., Scheifhackenf N. (2014). The long road to improving the water quality of the Western Bug River (Ukraine)—A multi-scale analysis. J. Hydrol..

[104] Odnorih Z., Manko R., Malovanyy M., Soloviy C. (2020). Results of Surface Water Quality Monitoring of the Western Bug River Basin in Lviv Region. J. Ecol. Eng..

[105] Andrzejewska A., Konarzewska J., Piątek M., Mikulski M. (2015). Environmental Protection Program for the Liw Commune for 2016–2019.

[106] Kluska M., Jabłońska J., Prukała W., Popiel S. (2021). Research on the Stability of Biologically Active (E)-azastilbene Derivatives in Polish Rivers. Pol. J. Environ. Stud..

[107] Jabłońska J., Kluska M. (2020). Determination of Mercury Content in Surface Waters Using an Environmentally Non-Toxic Terminating Electrolyte. Bull. Environ. Contam. Toxicol..

[108] Jabłońska J., Kluska M. (2019). Dynamics of mercury content changes in snow in the heating season on the example of the city of Siedlce. Ochr. Srodowiska I Zasobow Nat..

[109] Malá Z., Gebauer P., Boček P. (2017). Analytical capillary isotachophoresis after 50 years of development: Recent progress 2014–2016. Electrophoresis.

[110] Jabłońska J., Kluska M., Erchak N. (2020). The challenge of separating and determining biologically active electrostatically stabilized silanates using the high-performance liquid chromatography technique. J. Sep. Sci..

[111] Jabłońska J., Kluska M., Erchak N. (2018). Analytics of biologically active derivatives of electrostatically stabilized silanates by isotachophoresis. J. Liq. Chromatogr. Relat. Technol..

[112] Kluska M., Marciniuk-Kluska A., Prukala D., Prukala W. (2016). Analytics of Quinine and its Derivatives. Crit. Rev. Anal. Chem..

[113] Kluska M., Pypowski K., Chrząścik I., Erchak N. (2009). Isotachophoresis of Chosen Heptacoordinated Goshchava Silanates. J. Liq. Chromatogr. Relat. Technol..

[114] Prukała D., Chrząścik I., Prukała W., Pypowski K., Szymalska M., Kluska M. (2009). Isotachophoresis of Chosen Biologically Active (E)-Azastilbenes. J. Liq. Chromatogr. Relat. Technol..

[115] Kluska M., Pypowski K., Chrząścik I., Koval T., Erchak N. (2009). Separation and Determination of Chosen λ^5^-Silanates by an Isotachophoresis Technique. J. Liq. Chromatogr. Relat. Technol..

[116] Kluska M. (2008). Analytical Techniques in Determination of Biologically Active Organosilicons of the ES-Silanate Group. Crit. Rev. Anal. Chem..

[117] Kosobucki P., Buszewski B. (2010). Selected Imidazolium Ionic Liquids as a Terminating Electrolyte in Isotachophoresis. Anal. Lett..

[118] Kosobucki P., Buszewski B. (2008). Determination of tetrafluoroborate and chloride anions by capillary isotachophoresis and sup-pressed ion chromatography. Chem. Anal..

[119] Malá Z., Gebauer P. (2017). Capillary moving-boundary isotachophoresis with electrospray ionization mass-spectrometric detection and hydrogen ion used as essential terminator: Methodology for sensitive analysis of hydroxyderivatives of s -triazine herbicides in waters. J. Chromatogr. A.

[120] Malá Z., Gebauer P. (2019). Recent progress in analytical capillary isotachophoresis. Electrophoresis.

[121] Datinská V., Voráčová I., Schlecht U., Berka J., Foret F. (2018). Recent progress in nucleic acids isotachophoresis. J. Sep. Sci..

[122] Guo S., Jacroux T., Ivory C.F., Li L., Dong W.-J. (2019). Immunobinding-induced alteration in the electrophoretic mobility of proteins: An approach to studying the preconcentration of an acidic protein under cationic isotachophoresis. Electrophoresis.

[123] Hradski J., Duriš M., Szucs R., Moravský L., Matejcík Š., Masár M. (2021). Development of microchip isotachophoresis coupled with ion mobility spectrometry and evaluation of its potential for the analysis of food, biological and pharmaceutical samples. Molecules.

[124] Dobrowolska-Iwanek J., Jamka-Kasprzyk M., Rusin M., Paśko P., Grekh S., Jurczak A. (2023). Developed and validated capillary isotachophoresis method for the rapid determining organic acids in children’s saliva. Molecules.

[125] Jabłońska J., Kluska M., Erchak N., Prukała W. (2022). A non-toxic for environmental electrolyte terminating for the analysis of stilbene derivatives by the isotachophoresis technique. Int. J. Environ. Anal. Chem..

